# The ACS-NSQIP Analysis of Negative Pressure Wound Therapy Following Pancreatectomy for Pancreatic Diagnoses

**DOI:** 10.7759/cureus.59456

**Published:** 2024-05-01

**Authors:** Genaro DeLeon, Varun Rao, Ben Duggan, Timothy P Becker, Kevin Pei

**Affiliations:** 1 General Surgery, Indiana University School of Medicine, Indianapolis, USA; 2 Neurological Surgery, Indiana University School of Medicine, Indianapolis, USA; 3 General Surgery, Parkview Health, Fort Wayne, USA

**Keywords:** pancreas, negative pressure wound therapy, outcomes, pancreatectomy, acs-nsqip

## Abstract

Introduction

Surgical site infections (SSIs) continue to be a challenging issue among patients undergoing pancreatectomy. Anecdotally, the use of negative pressure wound therapy (NPWT) following pancreatectomy for cancer has been associated with decreased SSIs. The objective of this study was to compare the postoperative outcomes of NPWT and non-NPWT for incisional wound care following distal pancreatectomy or pancreatoduodenectomy for pancreatic diagnoses using a national surgical database.

Methods

The American College of Surgeons National Surgical Quality Improvement Program (ACS-NSQIP) was queried from 2005 to 2019 for patients undergoing distal pancreatectomy or pancreaticoduodenectomy for pancreatic diagnoses using primary Current Procedural Terminology (CPT) codes. The primary outcome was surgical site infection rates between NPWT and non-NPWT patient groups. Secondary outcomes include sepsis, septic shock, readmission, and reoperation. Outcomes of interest were compared using multivariate logistic regression.

Results

A total of 54,457 patients underwent pancreatectomy with 131 receiving NPWT. Multivariate analysis, while accounting for patient characteristics, including wound classification, showed no difference in postoperative superficial SSI, deep SSI, sepsis, septic shock, or readmission between the NPWT and non-NPWT groups. Organ space SSI was higher in the NPWT group (21% vs 12%, p=0.001). Reoperation related to procedure was also high in the NPWT group (14% vs 4.3%, p<0.001).

Conclusion

The use of NPWT in distal pancreatectomies and pancreatoduodenectomies is associated with increased organ space SSIs and reoperation rates, with no difference in superficial SSI, deep SSI, or readmission. This large sample study shows no significant benefit of using NPWT incisional wound care after pancreatectomy.

## Introduction

Surgical site infections (SSIs) continue to be a challenging issue among patients undergoing pancreatectomy. SSI is a common complication associated with pancreaticoduodenectomy that can lead to decreased disease-free and overall survival [1-3]. Morbidity, including wound infection, remains relatively high for this procedure which can also increase readmission rates [4]. Such complications following pancreatic resection burden the patient by increasing both the length of hospital stay and healthcare costs [5,6]. The overwhelming impact that SSIs have on patient readmission, survival, and postoperative hospital costs requires further investigation of interventions that can reduce SSI rates.

Poruk et al. developed a validated risk score to predict SSI through a retrospective review of 679 patients undergoing pancreaticoduodenectomy and found that application of a superficial vacuum closure, or negative pressure wound therapy (NPWT), led to a decrease in SSI rate compared to standard wound closure [7]. Subsequently, the same clinical group changed their clinical practice to include NPWT resulting in a decline of SSI rate, particularly for the highest-risk patients [8]. The use of NPWT to reduce SSIs is well documented in ventral hernia repairs and orthopedic surgery due to removal of excess fluid, increased blood flow, and decreased edema [9-12]. Despite these findings, the current literature on NPWT following pancreatectomy is limited to single-site studies with varying results leading to controversy over its benefits [7,8,13-16].

Negative pressure wound therapy (NPWT) operates through several molecular and physiological mechanisms that significantly influence wound healing and the prevention of surgical site infections (SSI). NPWT enhances wound healing by promoting angiogenesis, increasing blood flow, and reducing edema, thereby creating an environment conducive to tissue repair and regeneration. These effects are achieved through the application of controlled negative pressure, which also helps in the removal of excess fluids and infectious materials from the wound site [17]. Research by Norman et al. and Webster et al. corroborates the effectiveness of NPWT in reducing SSIs, highlighting its advantage for surgical wounds healing by primary closure through the optimization of the local wound environment [18,19]. Although specific studies on NPWT's role in pancreatic surgery are scarce, the general principles of enhanced wound healing and SSI prevention suggest its potential applicability. In pancreatic surgery, where the risk of postoperative complications and SSIs is significant due to the complexity of the procedures and the patient's condition, NPWT could offer a valuable adjunctive therapy to improve outcomes by leveraging its established mechanisms of action.

The objective of this study was to compare the postoperative outcomes of NPWT and non-NPWT for incisional wound care following distal pancreatectomy or pancreatoduodenectomy for pancreatic diagnoses using a large, national surgical database. We hypothesized that patients undergoing NPWT for incisional wound care would experience lower rates of SSIs compared to those undergoing non-NPWT.

## Materials and methods

Data source details

The American College of Surgeons National Surgical Quality Improvement Program (ACS-NSQIP) represents a comprehensive, prospective database designed to capture data from patients who have undergone surgical procedures across a wide network of over 570 participating hospitals, both community and university-based, within the United States. This database meticulously collects information on preoperative patient characteristics as well as outcomes occurring within 30 days postsurgery. For the purpose of this study, the utilization of ACS-NSQIP data received an exemption from review by the Parkview Institutional Review Board, underlining the database's established protocols for ensuring patient confidentiality and data integrity in research contexts.

Study design

This study embarked on a detailed analysis within the ACS-NSQIP database spanning from 2005 to 2019, focusing on patients who underwent either distal pancreatectomy (DP) or pancreaticoduodenectomy (PD), which are surgical procedures targeted at treating conditions of the pancreas. Identification of patients who underwent these procedures was systematically done using specific Current Procedural Terminology (CPT) codes: 48140 and 48146 for DP, and 48150, 48152, 48153, and 48154 for PD. Further, the study categorized patients based on whether they received a Negative Pressure Wound Therapy (NPWT) concurrently with their primary surgical procedure, with identification through CPT codes 97605, 97606, 97607, and 97608. The investigation primarily aimed to compare surgical site infection (SSI) rates between patients receiving NPWT and those who did not, alongside examining secondary outcomes such as sepsis, septic shock, hospital readmissions, and reoperations.

Statistical analysis approach

For the statistical analysis, R version 3.6.1 (Vienna, Austria: R Foundation), a well-regarded open-source software, was employed. Initial evaluations of demographic data were conducted using univariate analysis to establish basic understanding and differences across patient groups. The determination of statistical significance was grounded on a p-value threshold of less than 0.05. To delve deeper into the outcomes of interest - specifically, the rates of surgical site infections, sepsis, septic shock, readmissions, and reoperations - a multivariate logistic regression was applied. This approach allowed for the adjustment based on patient characteristics, thereby offering a more nuanced and accurate understanding of the impact of NPWT on postoperative outcomes. Importantly, the regression models incorporated over 20 risk factors recorded within the ACS-NSQIP database, ensuring a comprehensive effort to account for potential confounding variables that might influence the results.

## Results

A total of 54,457 patients were identified of which 54,326 underwent pancreatectomy alone and 131 received simultaneous NPWT. Table 1 shows the patient demographics and preoperative characteristics of the NPWT and non-NPWT groups. The average age was the same for the NPWT and non-NPWT groups, 50 years (p=0.5). A total of 56% of patients were female in the NPWT group compared to 51% in the non-NPWT group (p=0.2). Patients who underwent NPWT tended to be sicker. Rates of treated diabetes (27% vs 35%, p=0.027), renal failure (<0.1% vs 1.5%, p=0.008), and dyspnea (6% vs 9%, p=0.034) were significantly higher for patients who received NPWT. Patients in the non-NPWT group had lower ASA classification scores and rates of preoperative sepsis (p<0.001). Table 1 details the rates of ASA scores and types of preoperative sepsis between the NPWT and non-NPWT groups. Operative time was longer in the NPWT group compared to the non-NPWT group (400 vs 311 minutes, p<0.001). Total hospital length of stay was longer for the NPWT patients (11 days) compared to the non-NPWT patients (eight days) (p<0.001).

**Table 1:** Patient demographics, preoperative risk factors, and co-morbidities. NPWT: negative pressure wound therapy; hypertension med: hypertension requiring medication; CHF: congestive heart failure; COPD: chronic obstructive pulmonary disease; Preop sepsis: preoperative sepsis; SIRS: systemic inflammatory response syndrome; Optime: operative time; ASA: American Society of Anesthesiologists

Characteristic	Non-NPWT, N = 54,326	NPWT, N = 131	p-Value
Age	50 (41, 57)	50 (42, 57)	0.5
Sex			
Female	27,541 (51%)	74 (56%)	0.2
Male	26,765 (49%)	57 (44%)	
Race			
Black or African American	4,659 (8.6%)	16 (12%)	0.4
White	41,368 (76%)	102 (78%)	
Unknown/not reported	5,612 (10%)	7 (5.3%)	
Asian or Pacific Islander	2,020 (3.7%)	5 (3.8%)	
Native Hawaiian or Pacific Islander	82 (0.2%)	0 (0%)	
American Indian or Alaska Native	190 (0.3%)	1 (0.8%)	
Hispanic ethnicity	127 (0.2%)	0 (0%)	0.4
BMI			
Not obese (BMI≤30)	39,044 (72%)	90 (69%)	0.4
Obese (BMI≥30)	15,282 (28%)	41 (31%)	
Diabetes mellitus			
No	39,881 (73%)	85 (65%)	0.027
Insulin	6,913 (13%)	23 (18%)	
Non-insulin	6,573 (12%)	23 (18%)	
Oral medication	959 (1.8%)	0 (0%)	
Hypertension med	29,003 (53%)	63 (48%)	0.2
Smoking status			
Non-smoker	43,908 (81%)	97 (74%)	0.060
Smoker	10,417 (19%)	34 (26%)	
CHF			
No	54,134 (100%)	131 (100%)	>0.9
Yes	178 (0.3%)	0 (0%)	
Renal failure	54 (<0.1%)	2 (1.5%)	0.008
Dialysis	199 (0.4%)	0 (0%)	>0.9
Ascites	204 (0.4%)	2 (1.5%)	0.088
Dyspnea			
No	50,932 (94%)	119 (91%)	0.034
Moderate exertion	3,256 (6.0%)	10 (7.6%)	
At rest	138 (0.3%)	2 (1.5%)	
Steroid use	1,479 (2.7%)	4 (3.1%)	0.8
COPD	2,403 (4.4%)	6 (4.6%)	>0.9
Malnourishment	7,516 (14%)	21 (16%)	0.5
ASA classification			
1. Healthy	471 (0.9%)	0 (0%)	<0.001
2. Mild systemic disease	13,839 (25%)	18 (14%)	
3. Severe systemic disease	36,648 (67%)	96 (73%)	
4. Life threatening systemic disease	3,303 (6.1%)	15 (11%)	
5. Moribund	11 (<0.1%)	2 (1.5%)	
Bleeding disorder	1,646 (3.0%)	4 (3.1%)	>0.9
Blood transfusion	381 (0.7%)	9 (6.9%)	<0.001
Preop sepsis			
None	53,410 (98%)	124 (95%)	<0.001
Unknown	97 (0.2%)	0 (0%)	
Sepsis	222 (0.4%)	1 (0.8%)	
Septic shock	18 (<0.1%)	4 (3.1%)	
SIRS	579 (1.1%)	2 (1.5%)	
Total operation time (min.)	311 (222, 410)	400 (278, 515)	<0.001
Long optime			
Long (420+ min)	12,568 (23%)	58 (44%)	<0.001
Short (<420 min)	41,758 (77%)	73 (56%)	
Wound class			
1. Clean	3,198 (5.9%)	10 (7.6%)	0.002
2. Clean/contaminated	44,204 (81%)	90 (69%)	
3. Contaminated	5,628 (10%)	24 (18%)	
4. Dirty/infected	1,296 (2.4%)	7 (5.3%)	
Length of total hospital stay (days)	8 (6, 11)	11 (7, 19)	<0.001

Table 2 shows the postoperative outcomes between the NPWT and non-NPWT groups. There was no difference in postoperative superficial SSI, deep SSI, sepsis, septic shock, or readmission between the NPWT and non-NPWT groups. Organ space SSI was higher in the NPWT group (21% vs 12%, p=0.001). Any SSI was also higher in the NPWT group (31% vs 19%, p=0.001). Reoperation related to the procedure was also higher in the NPWT group (14% vs 4.3%, p<0.001).

**Table 2:** Postoperative outcomes between NPWT and non-NPWT following pancreatectomy for pancreatic indications using multivariate logistic regression. NPWT: negative pressure wound therapy; SSI: surgical site infection

Variables	Non-NPWT	NPWT	Odds ratio (95% CI)	p-Value
Complications	N=54,326	N=131		
Superficial incisional SSI	3,649 (6.7%)	14 (11%)	1.45 (0.82-2.55)	0.201
Deep incisional SSI	814 (1.5%)	5 (3.8%)	0.51 (0.20-1.34)	0.173
Organ/space SSI	6,315 (12%)	28 (21%)	2.03 (1.33-3.12)	0.001
Any SSI	10,090 (19%)	41 (31%)	1.86 (1.27-2.72)	0.001
Sepsis	4,261 (7.8%)	13 (9.9%)	1.16 (0.65-2.08)	0.621
Septic shock	1,481 (2.7%)	9 (6.9%)	1.66 (0.79-3.48)	0.183
Readmission related to procedure	6,173 (15%)	14 (14%)	0.89 (0.50-1.57)	0.686
Reoperation related to procedure	1,776 (4.3%)	14 (14%)	2.72 (1.51-4.89)	<0.001

## Discussion

In this retrospective cohort study, using a national surgical database, we found that simultaneous NPWT and pancreatectomy were associated with higher organ space SSIs and reoperation rates. However, there was no difference in superficial SSI, deep SSI, or readmission rates.

Cases in the NPWT cohort had approximately 9% higher organ space SSI rates than the non-NPWT cohort and were 3.3 times more likely to undergo a reoperation related to procedure. This contrasts with the existing literature, which shows both reduction of SSI after NPWT and no difference in SSI after NPWT. A 2019 single-center randomized, controlled trial found a significantly reduced risk of SSI with the use of NPWT in patients undergoing PD when compared to standard sterile dressing [13]. Several studies have found that NPWT does not improve SSI rates in the resection of pancreas neoplasms. A randomized trial conducted by Andrianello et al. revealed no differences in combined incisional SSI rates for periampullary neoplasms [14]. Smaller randomized trials have also found no difference in the rate of SSI when NPWT is used postoperatively for pancreatic resections; however, a possible explanation for no observed difference could be the duration of NPWT [15,16].

The reason for the lack of efficacy for NPWT in this clinical setting is unknown. One potential explanation is the different pathophysiology of postpancreatic neoplasm wound infections compared to other surgeries that showed increased efficacy of NPWT. Studies continue to show infectious complication rates between 10% and 20% following pancreatectomy [6,7]. This is lower than the incidence of wound infections in other surgeries where NPWT has been shown to decrease SSI. For example, in ventral hernia repair and ventral hernia repair with concurrent panniculectomy, the wound infection rates are as high as 40% and 70%, respectively [10,11]. Since the incidence of SSI is already lower in pancreatectomy operations, it could be that patients experience a less robust response to NPWT compared to surgeries such as ventral hernia repair. In addition, ventral hernia repairs such as those with concurrent panniculectomy involve lipocutaneous flaps that are poorly vascularized resulting in worse morbidity, highlighting a difference in pathophysiology between surgeries where NPWT may have different effects on SSI [10]. A similar explanation regarding pathophysiology could be given for the success of NPWT in orthopedic trauma surgery, where the incidence of SSI after lower-extremity fractures is similar to pancreatectomy at 19% [12]. In orthopedic trauma cases the mechanism of the surgical indication, high-energy trauma, can contribute to challenging wound healing requiring higher level wound care in the form of NPWT.

An optimal duration of NPWT application to primary abdominal incisions is not yet defined [16]. This could be the reason we did not observe any differences in superficial SSI or deep SSI; however, the database does not contain the granularity to determine NPWT duration. To the best of our knowledge, this is the first study comparing postoperative outcomes of NPWT and non-NPWT for incisional wound care following distal pancreatectomy or pancreatoduodenectomy using a large national database. Previous studies have consisted of single-site patient populations, and results have varied [13-16].

This study had several limitations. The retrospective nature of the database may introduce selection bias. The study was also limited by the available data points recorded in the database as well as operative details such as surgeon decision-making or operative experience. These factors are difficult to record in a retrospective database, so whether surgeons chose to perform NPWT for more complicated cases could not be elucidated. In addition, our study could not account for several important variables that significantly impact surgical outcomes due to their absence in the ACS-NSQIP database. Notably, information on preoperative biliary stenting, a well-known risk factor for surgical site infection (SSI) in pancreatoduodenectomy; the use of wound protectors; postoperative complication grades using the standard Clavien-Dindo classification; and the occurrence of postoperative pancreatic fistula or bile leak, which are common and clinically significant events in pancreatic surgery, were not available for analysis. We were also unable to delve into the different types of NPWT used, as the ACS-NSQIP does not provide these nuances. Our study only evaluated 30-day outcomes due to the limited follow-up of the ACS-NSQIP database. Since long-term postoperative outcomes were not available, 90-day morbidity and mortality could not be analyzed. Lastly, this study did not use propensity score matching or inverse probability weighting to adjust for confounders, potentially affecting the observed associations.

## Conclusions

The use of NPWT in distal pancreatectomies and pancreatoduodenectomies is associated with increased organ space SSIs and reoperation rates, with no difference in superficial SSI, deep SSI, or readmission. This large sample study shows no significant benefit with the use of NPWT incisional wound care after pancreatectomy. Further multisite prospective studies are needed to assess the outcomes of simultaneous pancreatectomy and NPWT.
